# Using Sexual Selection Theories to Examine Contextual Variation in Heterosexual Women’s Orientation Toward High Heels

**DOI:** 10.1007/s10508-019-01539-3

**Published:** 2019-09-16

**Authors:** Christopher Watkins, Amanda Leitch

**Affiliations:** Division of Psychology, School of Social and Health Sciences, Abertay University, Bell Street, Dundee, DD11HG Scotland

**Keywords:** Sartorial appearance, Fashion, Footwear, Sex drive, Sexual selection

## Abstract

High heels are symbols of female sexuality and are “costly signals” if the risks of wearing them are offset by improving women’s attractiveness to men. From a functionalist perspective, the costs versus benefits of wearing heels may vary according to personal and contextual factors, such as her effectiveness at competing for mates, or at times when such motives are stronger. Here, we examined potential differences between women (self-rated attractiveness, dyadic versus solitary sexual desire, women’s age, competitive attitudes toward other women) and contextual variation (priming mating and competitive motives) in their responses to high heels. Study 1 (*N* = 79) and Study 2 (*N* = 273) revealed that self-rated attractiveness was positively related to orientation toward heeled shoes. When examining responses to two very attractive shoes (one higher heel, one lower heel) in Study 2, dyadic sexual desire, but not solitary sexual desire or intrasexual competitiveness, predicted their inclination to buy the higher-heeled shoe. In Study 3 (*N* = 142), young women chose high heels when primed with free choice of a designer shoe (95% CI [53.02 mm, 67.37 mm]) and preferred a heel 22 mm (0.87”) higher than older women (Study 4, *N *= 247). Contrary to predictions, priming mating or competitive motives did not alter women’s preference toward a higher heel (Studies 3 and 4). Our studies suggest that attractive women augment their physical appeal via heels. High heels may be a subtle indicator of dyadic sexual desire, and preferences for heels are stronger at times in the lifespan when mating competition is relatively intense.

**Electronic supplementary material:**

The online version of this article (10.1007/s10508-019-01539-3) contains supplementary material, which is available to authorized users.

## Introduction

One form of female sartorial appearance is her choice of footwear. People have worn heeled shoes for over 500 years, with their use extended across different socioeconomic groups in the late nineteenth century (Linder & Saltzman, 1998; see also Parmentier, 2016; Semmelhack, 2008). In modern culture, high heels are a symbol of female sexuality and power (Dietz & Evans, 1982; Small, 2014; Smith, 1999). However, women who wear heels may incur costs. For example, heels contribute, in part, to osteoarthritis among women by increasing the force applied across the patellofemoral joint and the compressive force on the medial compartment of the knee (Kerrigan, Todd, & Riley, 1998), with these negative effects strengthened as the weight of the wearer increases (Titchenal, Asay, Favre, Andriacchi, & Chu, 2015). Moreover, biomechanical studies demonstrate that discomfort is related to heel height, as higher heels shift pressure onto different areas of the foot (Hong, Lee, Chen, Pei, & Wu, 2005). Recent systematic reviews demonstrate that high heels contribute to hallux valgus, musculoskeletal pain, and risk of injury while walking (reviewed in Barnish & Barnish, 2016). Collectively, people who wear high heels may incur costs to health.

Evolutionary perspectives to consumer behavior (see Durante & Griskevicius, 2016; Saad, 2013) generate predictions based on the potential functional benefits of purchasing and, in turn, displaying various items to onlookers (e.g., Hill, Rodeheffer, Griskevicius, Durante & White, 2012). Indeed, various items of apparel and makeup alter judgments of traits that may be important for human mate choice (see Rowland & Burriss, 2017 for a recent review pertaining to color). Consistent with theoretical accounts of costly signals (Zahavi, 1975) in consumer behavior (Miller, 2009; Saad, 2011, 2013), high heels may be conceptualized as a costly signal if the risks associated with their use (Barnish & Barnish, 2016) are offset by promoting one’s attractiveness to observers, an important component of female intrasexual competition (reviewed in Vaillancourt, 2013). Consistent with this proposal, biological motion research demonstrates that high heels, compared to flat shoes, enhance the attractiveness of a woman’s walk by accentuating attractive and feminine bodily features related to gait such as smaller and more frequent steps, greater pelvic rotation, and pelvic tilt (Morris, White, Morrison, & Fischer, 2013). Moreover, men may be able to detect these changes to women’s attractiveness, as they appear to display more prosocial behavior toward women in heeled shoes (Guéguen, 2015). In sum, limited experimental work to date suggests that high heels may be a cultural aid for successful mate choice and mating-related competition, by enhancing female attractiveness to observers.

Here, across four studies, we report the results of research examining possible individual and contextual variation in women’s responses to heeled shoes (higher- versus lower-heeled shoes). First, although self-promotion and appearance enhancement are important in female competition for mates (Vaillancourt, 2013) and high heels enhance female attractiveness (Morris et al., 2013), it is unclear whether wearing high heels functions either to augment or to compensate for women’s physical attractiveness (i.e., in light of her own attractiveness). Thus, we tested for positive versus negative relationships between women’s self-rated attractiveness (Lynn, 2009; Weeden & Sabini, 2007; see also Little, Jones, & DeBruine, 2011) and her preference for higher-heeled versus lower-heeled shoes, when carefully controlling for differences in attractiveness between a set of images of higher-heeled and lower-heeled shoes. Evidence that high heels function to augment female attractiveness would be consistent with our proposal that high heels are a costly signal (Miller, 2009; Saad, 2011, 2013), because they are preferred by women who can maximize the benefits and offset the potential costs to health (Barnish & Barnish, 2016) from wearing them (i.e., effective competitors for mates; Vaillancourt, 2013). Alternately, evidence that less attractive women have a stronger preference for high-heeled shoes would speak to the importance of self-promotion and appearance enhancement in attractiveness-based competition among women (Vaillancourt, 2013), as it would suggest that women use this form of cultural apparel to improve their appearance when competing for mates.

Secondly, we examined potential contextual variation in women’s preferences for higher- versus lower-heeled shoes by testing whether sexual desire and chronological age are related to women’s preference for higher-heeled shoes. Although heels are a cultural symbol of female sexuality (Small, 2014), evidence that inclinations to buy higher-heeled shoes are related to dyadic sexual desire but not solitary sexual desire would provide the first empirical evidence (to our knowledge) that wearing high heels acts as a (subtle) cue to female sexual motivation. With age, as female mating competition is more intense during adolescence and early adulthood (reviewed in Vaillancourt, 2013), we predicted a negative relationship between women’s own age and their orientation toward heeled shoes, if women gain the greatest benefits from these “costly signals” (Barnish & Barnish, 2016; see Miller, 2009; Saad, 2011, 2013) in contexts where mating competition is relatively intense.

Finally, using experimental priming techniques, we report the results of two studies designed to test whether general preference for heel height can alter within women in contexts designed to prime mating motivations and competitive motivations (Griskevicius et al., 2009). Motivations that are important from the perspective of evolutionary biology have downstream effects on cognition, behavior (Kenrick, Neuberg, Griskevicius, Vaughn Becker, & Schaller, 2010; Maner & Ackerman, 2015), and consumer choice (Durante & Griskevicius, 2016; Miller, 2009; Saad, 2011, 2013). Thus, we predicted that women would enhance their attractiveness (Morris et al., 2013) by preferring higher heels in contexts where the benefits to wearing them are greater and their associated costs to health (Barnish & Barnish, 2016) are reduced (i.e., because mating competition is relatively intense). In two studies, we also tested for correlational relationships between women’s scores on a questionnaire measure of intrasexual competitiveness and their responses to heeled shoes. Collectively, evidence that mating and/or competitive motives are related to preferences for greater heel height would suggest that circumstantial factors related to intersexual and intrasexual selection alter women’s orientation toward one form of cultural apparel.

## Study 1

### Method

#### Participants

A total of 94 women took part in our laboratory study (*M*_age_ = 23.20 years, SD= 6.40 years; three participants did not provide their age). Participants were recruited via word of mouth, campus Intranet, and our research participation scheme. All participants were entered into a prize draw for a £10 shopping voucher, except for those who received course credit via our research participation scheme. Data were analyzed for heterosexual women only, and data from two participants were excluded for misunderstanding the instructions for the shoe preference task (*N* = 79: *M*_age_ = 23.49 years, SD= 6.80 years; two participants did not provide their age). All procedures for recruitment and testing (reported throughout) were approved by our local ethics committee. All participants gave informed consent before taking part.

#### Measures

Sixty images of shoes were extracted via screenshot from an online shoe retailer (kurtgeiger.com) who provide statistics for all of their items of footwear (including cost, measured heel height, color, material). Images were downloaded in August 2016. Each shoe was centered onscreen via PowerPoint and then saved as a jpeg file before cropping via editing software (GIMP v2.6.7) to 600 × 600 pixels. Because color cues alter various trait judgments of clothing and apparel (Guéguen, 2012; see Rowland & Burriss, 2017 for a recent review), these cues were removed via the program’s desaturated luminosity function (gray level calculated as: luminosity = 0.21 × *R* + 0.72 × *G* + 0.07 × *B*). A total of 85 participants (13 of whom were male, *M*_age_ = 31.86 years, SD= 10.72 years) rated a subset of 20 out of 60 shoes in a randomized order on surveymonkey.com on the attributes “attractive,” “practical,” “sexy,” “comfortable,” “stylish,” “has a high heel,” “fashionable,” and “expensive,” on a 1 (not at all) to 7 (very) scale. Each shoe received between nine and 34 ratings. Agreement between participants who rated all of their allocated set of shoes was high (all Cronbach’s alpha > .87 and < .94). Agreement between raters was also good when rating shoe attractiveness (all Cronbach’s alpha > .70 and < .85).

Across all 60 shoes, all trait ratings were correlated with one another (all *p *< .01, see Table 1), except for nonsignificant correlations between actual cost and judgments of attractiveness, stylishness, and fashionable (all rho< .20, all *p *> .12). Actual cost (statistics not reported in table) was negatively correlated with judgments of practicality (rho= − .54; *p *< .001) and comfortable (rho= − .55; *p *< .001) and was correlated with judgments of sexiness (rho= .36; *p *< .01) and heel height (rho= .52; *p *< .001). Of note, when examined against the data provided by the Web site, participants could accurately judge expensiveness (rho= .41; *p *= .001) and heel height (rho_59_ = .96; *p *< .001).

**Table 1:** Correlations for trait ratings of full sample of shoes

	Practical	Sexy	Comfortable	Stylish	Heel	Fashionable	Expensive
Attractiveness	− .53	.92	− .59	.88	.63	.85	.70
Practical		− .75	.97	− .35	− .88	− .42	− .61
Sexy			− .79	.79	.79	.79	.74
Comfortable				− .39	− .89	− .46	− .64
Stylish					.47	.92	.70
Has a high heel						.55	.70
Fashionable							.70
Expensive							

All correlations reported here are significant at *p *< .01

From these data, we selected seven shoes with a higher heel and seven shoes with a lower heel. The higher-heeled shoes were matched in attractiveness (*M *= 4.09, SD= 0.92) to the lower-heeled shoes (*M *= 3.82, SD= 0.53; *t*[12] = .67; *p *= .51). Critically, the two sets of shoe images differed on both perceived heel height and actual heel height when comparing the higher-heeled image set (*M*_Perceived_ = 6.44, SD= 0.29; *M*_Actual_ = 121 mm, SD= 20 mm) to the lower-heeled image set (*M*_Perceived_ = 4.19, SD= 0.62; *M*_Actual_ = 75 mm, SD= 10 mm, both *t *> 5.38; both *p *< .001). Effect sizes: *r* = .84 (perceived height) and *r* = .95 (measured height).

#### Procedure

Participants first took part in a shoe preference task run on SuperLab version 4.5. Here, participants viewed 14 randomized trials (seven higher-heeled shoes, seven lower-heeled shoes) for 3 s each with a fixation-cross presented for 500 ms in between each trial. Participants were informed that they would be shown a slideshow of various items of footwear and that on each trial they should indicate whether they would buy the item of footwear with a yes (y) or no (n) key press. Participants were informed that they had 3 s to make a decision on each trial, at which point the slideshow would move on to the next pair of shoes. Participants were asked to place a finger by the “y” and “n” keys for the duration of the task.

Following this, participants reported their self-rated attractiveness on a 1 (much less attractive than average) to 7 (much more attractive than average) scale, their height in centimeters, and completed the Sexual Desire Inventory II (Spector, Carey, & Steinberg, 1996, *M*_DyadicDesire_ = 40.25, SD= 10.55, range = 13–66; *M*_SolitaryDesire_ = 9.22, SD= 5.50, range = 3–23) and the Intrasexual Competitiveness Questionnaire (Buunk & Fisher, 2009*M*_Score_ = 2.50, SD= 0.98, Range = 1.00–4.73). The 14-item Sexual Desire Inventory II contains eight items measuring dyadic sexual desire and three items measuring solitary sexual desire, with high scores indicating high desire (possible range of scores is 8–70 for dyadic sexual desire and 3–26 for solitary sexual desire). Each subscale has very good internal consistency (Spector et al., 1996) with scores on each subscale correlating with neural activity in response to viewing sexual images (Demos, Heatherton, & Kelley, 2012). The Intrasexual Competitiveness Questionnaire has high reliability and excellent measurement equivalence across different language versions of the questionnaire (Buunk & Fisher, 2009) and consists of 12 items rated on a 1 (not at all applicable) to 7 (completely applicable) scale, with high scores indicating high competitiveness. Self-rated attractiveness is correlated with objective measures of attractiveness and attractiveness ratings of face photographs (Weeden & Sabini, 2007) and prosocial biases toward attractive individuals in naturalistic contexts (e.g., tipping; Lynn, 2009). Questionnaires were completed in a randomized order. Participants were then debriefed and thanked for participation.

#### Data Analysis

For each participant, we calculated, separately, the proportion of trials on which they chose a higher-heeled shoe and the proportion of trials on which they chose a lower-heeled shoe. Trials on which participants did not respond (1.32% of trials across the whole sample) were not included in analyses. Removing these trials versus recoding missing responses as zero did not alter any of the conclusions from analyses reported here. Scores could thus range from zero to one, with high scores indicating a stronger preference for selecting a given type of shoe.

### Results

In an initial analysis to examine how selective our sample were, one-sample *t* tests against chance (i.e., 0.5) revealed that women chose the shoes from our higher-heeled image set (*M *= .42, SD= .25) and lower-heeled image set (*M *= .36, SD= .21) at levels less than would be expected by chance (i.e., they were generally selective; both absolute *t *> 2.92, both *p *< .01).

Next, a within-subjects ANCOVA was conducted on the dependent variable preference for shoes, with the factor shoe type (higher-heeled shoe, lower-heeled shoe) and the covariate self-rated attractiveness. This analysis revealed an effect of shoe type that approached significance (*F*[1, 77] = 3.47; *p *= .07, np^2^ = .04) and an interaction between shoe type and self-rated attractiveness (*F*[1, 77] = 6.67; *p *= .012, np^2^ = .08). There was no main effect of self-rated attractiveness (*F*[1, 77] = 1.16; *p *= .29). Rerunning the ANCOVA with additional covariates in our model (participant age, participant height, dyadic sexual desire, solitary sexual desire, intrasexual competitiveness score) revealed no further significant effects or interactions (all *F *< 2.38, all *p *> .12). In this model, our two-way interaction remained significant (*F*[1, 65] = 6.60; *p *= .013, np^2^ = .09).

**Fig. 1:**
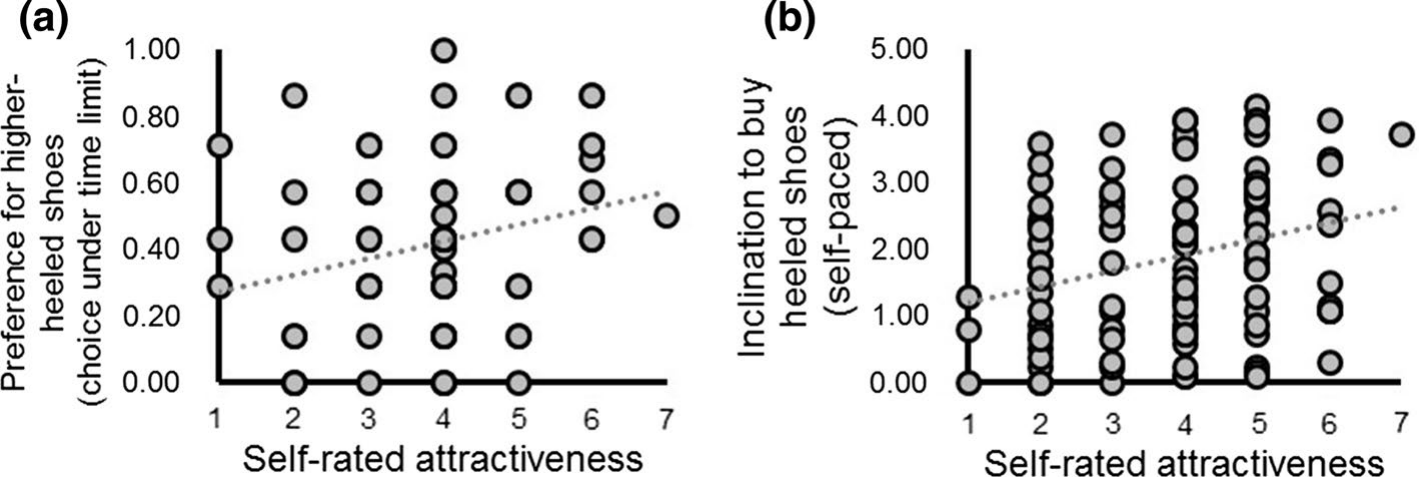
Relationships between self-rated attractiveness and women’s preference for heeled shoes. Panel **a**. Self-rated attractiveness is correlated with preference for higher-heeled shoes on time-limited trials (rho[70] = .24). Panel **b**. Self-rated attractiveness is correlated with women’s inclination to buy a heeled shoe in a self-paced task (*N* = 119, np^2^ = .08)

Correlational tests were conducted to interpret the interaction between shoe type and self-rated attractiveness. While self-rated attractiveness was positively correlated with women’s preference for higher-heeled shoes on time-limited trials (*rho*[79] = .24; *p *= .033, Fig. 1a), it was not correlated with women’s preference for lower-heeled shoes on time-limited trials (*rho*[79] = − .10; *p *= .38). The slopes of these two correlations differed significantly from one another (*Z *= 2.40, *p *= .02). A regression analysis on the predictor variable self-rated attractiveness and the outcome variable preference for higher-heeled shoes revealed a significant model (*F*[1, 77] = 5.11; *p *= .027) with the predictor variable explaining 6.2% of the variance in the outcome variable (adjusted *R*^2^ = .05, standardized beta = .25, *t *= 2.26; *p *= .027).

## Study 2a

### Method

#### Participants

A total of 125 heterosexual women (*M*_age_ = 24.36 years, SD= 3.08 years; three women did not report age) took part in an online study hosted on surveymonkey and distributed via prolific academic (prolific.ac). We set recruitment criteria of women aged 18–30 years whose first language was English, with participants reimbursed the equivalent of just above £5 per hour. Online and laboratory studies produce equivalent results (reviewed in Gosling, Vazire, Srivastava, & John, 2004), and online methods have been used in past research on women’s consumer behavior (Hudders, De Backer, Fisher, & Vyncke, 2014). The platform we used generates reliable data (Peer, Brandimarte, Samat, & Acquisti, 2017).

#### Procedure

Participants were recruited for a study on fashion, appearance, and sexuality. Women provided demographic information, and self-rated attractiveness as measured in Study 1. In the main task, women were told that they would take part in a consumer decision-making task and that on each trial they would be asked, based on their first impressions, whether they would buy the pictured item of footwear. They were reminded that we were interested in their first impressions and they did not have to spend too long on a given trial. Participants rated the identical 14 shoes used in Study 1 in a randomized order, with the instructions “Based on your first impressions, use the scale below to indicate your feelings on whether you would buy this item.” On each trial, participants rated the item on an 8-point scale from “would not buy” to “would definitely buy” (responses coded from 0 to 7). Following this task, participants completed the Sexual Desire Inventory (Spector et al., 1996, *M*_Dyadic Desire_ = 39.31, SD= 13.51, Range = 8–66; *M*_Solitary Desire_ = 12.50, SD= 6.65, Range = 3–25) and were debriefed online. Reliability on this questionnaire was very high in the current study (Cronbach’s alpha: dyadic sexual desire = 0.91; solitary sexual desire = 0.91).

#### Data Analysis

We used these data to calculate, across trials, women’s inclination to buy heeled shoes, separately for the higher-heeled image set and the lower-heeled image set. High scores indicate a stronger inclination to buy a given set of heeled shoes.

### Results

Initial one-sample *t* tests against chance (i.e., 3.5) revealed that women, on average, were inclined to buy shoes within the higher-heeled set (*M *= 1.72, SD= 1.40) and lower-heeled set (*M *= 2.04, SD= 1.33) at levels *less* than would be expected by chance (both absolute *t*[118] > 12.01, both *p *< .001).

A within-subjects ANCOVA on the dependent variable inclination to buy shoes, with the factor shoe type (higher-heeled shoe, lower-heeled shoe) and the covariate self-rated attractiveness revealed no effect of shoe type or higher-order interaction between shoe type and self-rated attractiveness (both *F *< .39, both *p *> .53). A main effect of self-rated attractiveness was observed (*F*[1, 117] = 9.87; *p *< .01, np^2^ = .08). Regression analysis on the predictor variable self-rated attractiveness and the outcome variable inclination to buy shoe (collapsed across shoe type) revealed a significant model (*F*[1, 117] = 9.87; *p *< .01), where self-rated attractiveness explained 7.8% of the variance in the outcome variable (adjusted *R*^2^ = .07, standardized beta = .28, *t *= 3.14; *p *< .01, see Fig. 1b).

Rerunning the ANCOVA with the inclusion of additional covariates participant age, dyadic sexual desire and solitary sexual desire revealed the same main effect of self-rated attractiveness (*F*[1, 104] = 7.62; *p *< .01, np^2^ = .07). No other effects or interactions in the model were significant (all *F *< 2.27, all *p *> .13), except for an interaction between shoe type and participant age that approached significance (*F*[1, 104] = 3.87; *p *= .052, np^2^ = .04).

As both studies suggest that women were selective in choosing shoes when analyzing responses to an entire image set, individual differences in preferences and/or choices may also be observed when examining responses to specific items (e.g., at the extremes of attractiveness within an image set). In light of this, we conducted a further analysis on women’s responses to two attractive shoes within the set. Based on pilot data (see Table 2 for descriptive statistics), we selected the most attractive lower-heeled shoe (pilot data: 1.44 SD above the mean in rated attractiveness) and the most attractive higher-heeled shoe in the set that did not differ in attractiveness from the lower-heeled shoe (pilot data: 2.20 standard deviations above the mean in rated attractiveness, *t* test, *p *= .15). In the pilot study, only one higher-heeled shoe used in this study had a greater mean attractiveness rating (*M *= 5.30) than the higher-heeled shoe used in this follow-up analysis. In our sample for Study 2a, these two shoes were the most popular items out of all 14 shoes in the image set (*M*_Lower-heeled_ = 3.27, SD= 2.05, 2.11 SD above the mean of the set; *M*_Higher-heeled_ = 2.65, SD= 2.17, 1.19 SD above the mean of the set).

**Table 2:** Descriptive statistics (*M* and SD) for the two attractive items of footwear (pilot data)

	Higher-heeled shoe *M* (SD)	Lower-heeled shoe *M* (SD)
Attractiveness	5.24 (1.26)	4.57 (1.79)
Practical	2.19 (1.21)	2.87 (1.74)
Sexy	5.62 (1.43)	4.45 (1.71)
Comfortable	2.52 (1.17)	3.20 (1.65)
Stylish	5.43 (1.16)	4.77 (1.74)
Perceived heel height	6.05 (0.86)	4.70 (1.62)
Fashionable	5.52 (0.98)	4.77 (1.65)
Expensive	4.71 (1.49)	4.00 (1.44)
Measured heel	115 mm	85 mm

#### Additional Analyses: Two Attractive and Popular Items of Footwear

Initial one-sample *t* tests against chance (i.e., 3.5) revealed that, on average, women’s inclination to buy the lower-heeled attractive shoe (*M *= 3.27, SD= 2.05) did not differ from chance (absolute *t*[124] = 1.24; *p *= .22). Women’s inclination to buy the higher-heeled attractive shoe (*M *= 2.65, SD= 2.17) was significantly less than would be expected by chance (absolute *t*[123] = 4.34; *p *< .001).

A repeated-measures ANCOVA was conducted on the dependent variable inclination to buy the shoe, with the within-subjects factor shoe (attractive higher-heeled item, attractive lower-heeled item) and the covariate self-rated attractiveness. This analysis revealed an effect of self-rated attractiveness that approached significance (*F*[1, 122] = 3.34; *p *= .07, np^2^ = .03). No other effects or interactions were significant (both *F *< 2.64, both *p *> .10). Of note, this indicates that participants did not differ in their response to the two shoes when analyzing data via this model (i.e., there was a null effect of shoe).

Next, the ANCOVA was rerun with the within-subjects factor shoe (attractive higher-heeled item, attractive lower-heeled item) and the covariates self-rated attractiveness, dyadic sexual desire, solitary sexual desire, and participant age. This analysis revealed a significant interaction between shoe and dyadic sexual desire (*F*[1, 108] = 11.45; *p *= .001, np^2^ = .10). A main effect of solitary sexual desire was significant (*F*[1, 108] = 4.10; *p *= .045, np^2^ = .04) and an effect of self-rated attractiveness approached significance (*F*[1, 108] = 3.51; *p *= .064, np^2^ = .03). The former effect of solitary sexual desire reflected a nonsignificant positive correlation between solitary sexual desire and inclination toward buying the two items of footwear (*rho*[116] = .15; *p *= .11). No other effects or interactions were significant (all *F *< 2.53 all *p *> .11).

**Fig. 2:**
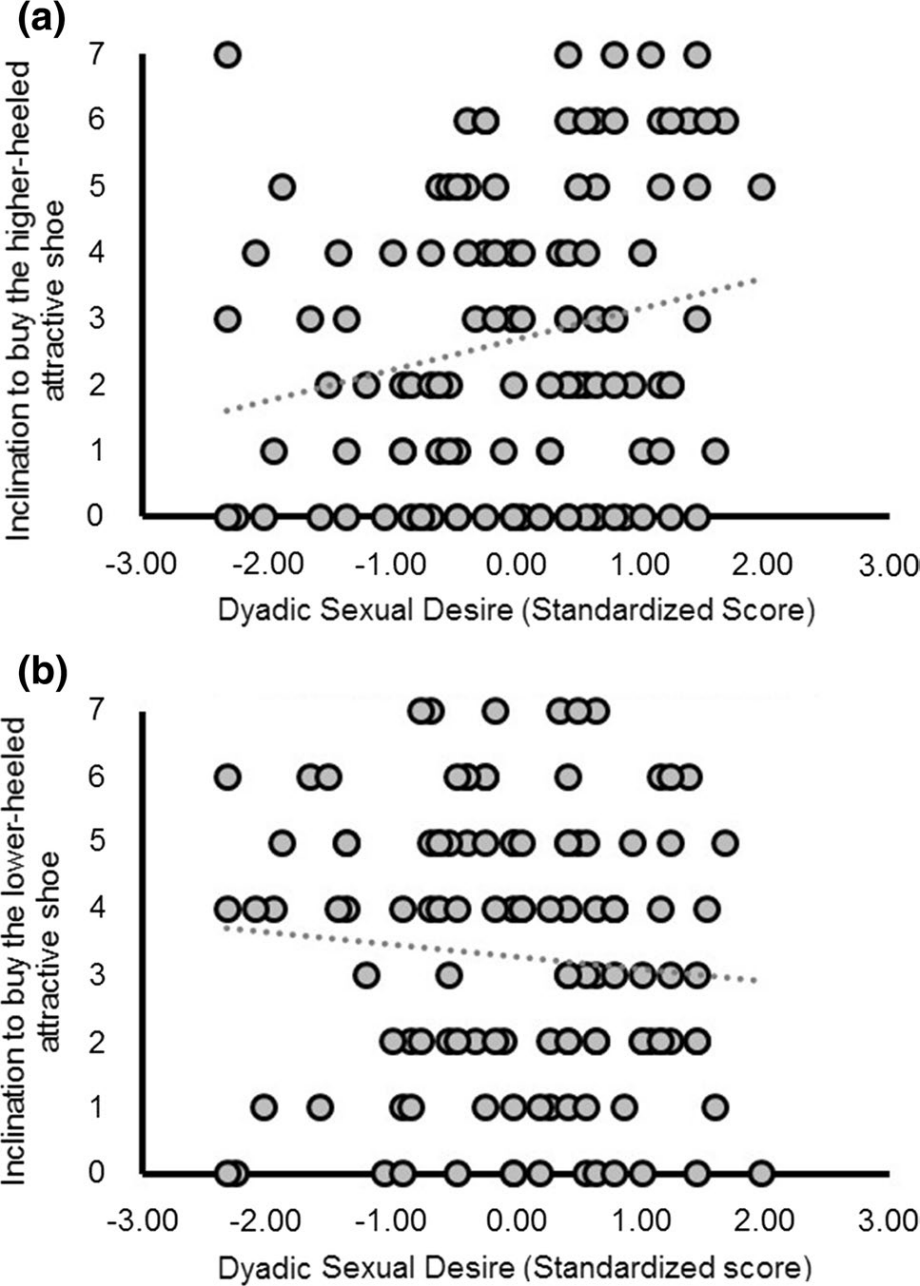
Dyadic sexual desire is related to women’s inclination to buy an attractive higher-heeled shoe (Panel **a**) but is not related to their inclination to buy an attractive lower-heeled shoe (Panel **b**, np^2^ = .10). The slopes of these two correlations differed significantly from one another (*N* = 116, *Z *= 3.62, *p *< .001)

Follow-up correlational tests were conducted to interpret the two-way interaction (*N* = 116 due to missing responses by some participants to the SDI questionnaire). These analyses revealed that women’s inclination to buy the higher-heeled attractive shoe was stronger as dyadic sexual desire increased (rho[116] = .22; *p *= .02), but dyadic sexual desire was not related to their inclination to buy the lower-heeled attractive shoe (rho[116] = − .11; *p *= .25). The slopes of these two correlations differed significantly from one another (*Z *= 3.62, *p *< .001, see Fig. 2).

## Study 2b

### Method

#### Participants

A total of 148 heterosexual women (*M*_age_ = 26.59 years, SD= 3.17 years; one woman did not report age) were recruited and took part in an online study on shoes, appearance, and social interaction. Participants were not eligible to take part in this study if they participated in Study 2a.

#### Procedure

The study was identical to Study 2a, except that women completed the Intrasexual Competitiveness Questionnaire (Buunk & Fisher, 2009; *M*_Score_ = 2.48, SD= 1.25, range = 1.00–7.00) after rating the shoes and did not complete the SDI-II. Reliability on this questionnaire was very high (Cronbach’s alpha = 0.92).

### Results

Initial one-sample *t* tests against chance (i.e., 3.5) revealed that women, on average, were inclined to buy shoes within the higher-heeled set (*M *= 1.99, SD = 1.61) and lower-heeled set (*M *= 1.85, SD= 1.26) at levels less than would be expected by chance (both absolute *t*[147] > 11.37, both *p *< .001).

A within-subjects ANCOVA on the dependent variable inclination to buy shoes, with the factor shoe type (higher-heeled shoe, lower-heeled shoe) and the covariate self-rated attractiveness revealed no effects or higher-order interaction between shoe type and self-rated attractiveness (all *F *< 2.59, all *p *> .11). Rerunning the ANCOVA with the additional covariates participant age and intrasexual competitiveness score revealed no significant effects or interactions (all *F *< 2.31, all *p *> .13) except for an effect of self-rated attractiveness that would be significant in a one-tailed test (*F*[1, 139] = 2.92; two-tailed *p* = .09, np^2^ = .02). We consider one-tailed *p* values in this instance in light of significant relationships between self-rated attractiveness and women’s responses to heeled shoes in our prior studies. Pooling data across Studies 2a and 2b revealed a significant correlation between self-rated attractiveness and women’s inclination to buy heeled shoes (standardized beta = .19, 95% CI [.07, .27], *t *= 3.19; *p *< .01). In this pooled regression analysis, the model was significant (*F*[1, 265] = 10.19; *p *< .01) and the predictor variable explained 3.7% of the variance in the outcome variable (adjusted *R*^2^ = 3.3%).

Next, we analyzed responses to the same two pairs of attractive shoes as analyzed in Study 2a. (The current sample also responded most positively toward these two items.) On average, women’s inclination to buy these two pairs of shoes (*M*_higher heel_ = 2.93, SD= 2.36, *M*_lower heel_ = 2.84, SD= 2.32) was significantly less than would be expected by chance (both absolute *t *> 2.92, both *p *< .01). An ANCOVA on women’s tendency to buy the shoe, with the factor shoe (attractive higher-heeled item, attractive lower-heeled item) and the covariate self-rated attractiveness, revealed an effect of shoe (*F*[1, 146] = 4.17; *p *= .043, np^2^ = .03) that was qualified by an interaction with self-rated attractiveness (*F*[1, 146] = 4.01; *p *= .047, np^2^ = .03). There was no main effect of self-rated attractiveness (*F*[1, 146] = 2.22; *p *= .14). Correlational tests to interpret our two-way interaction revealed that attractive women were more inclined to buy the attractive lower-heeled shoe (rho[148] = .21; *p *< .01), but self-rated attractiveness was not related to women’s inclination to buy the attractive higher-heeled shoe (rho[148] = .03; *p *= .70). The slopes of these two correlations differed significantly from one another (*Z *= 2.11; *p *= .03).

Inclusion of additional covariates in this model (participant age, intrasexual competitiveness score) revealed a main effect of participant age (*F*[1, 139] = 5.43; *p *= .021, np^2^ = .04). An interaction between shoe and self-rated attractiveness approached significance (*F*[1, 139] = 3.51; *p *= .063, np^2^ = .03) and an interaction between shoe and intrasexual competitiveness score also approached significance (*F*[1, 139] = 3.73; *p *= .056, np^2^ = .03). No other effects or interactions were significant (all *F *< 2.00 all *p *> .16). Correlational tests to interpret the main effect of participant age revealed that, across the two pairs of attractive heeled shoes, older women within our 18- to 30-year-old sample were more inclined to buy the attractive heeled shoes than younger women (rho[147] = .20, *p *= .017).

## Study 3

### Method

#### Participants

A total of 142 heterosexual women (*M*_age_ = 23.78 years, SD= 2.91 years, one woman did not provide her age) took part in the study hosted on surveymonkey.com. Participants (whose first language was English) were recruited via prolific academic and were reimbursed at a rate equivalent to just above £5 per hour. Prior work on consumer behavior demonstrates that priming experiments provide converging evidence when conducted online and in the laboratory (Jiang, Zhan, & Rucker, 2014).

#### Procedure

Participants were told that they would be asked to read a story depicting a scenario, which they may or may not be asked questions about later, and that they would be asked to complete a short task about shoe preferences. Following Griskevicius et al. (2009), participants were randomly allocated to one of three conditions used to prime one of three motives (mating motives, competitive motives, general arousal). For all primes, participants were asked to read the following scenario and as they read the scenario to try to put themselves in the shoes of the main character and experience the emotions that they were feeling. Participants either read a short story about a romantic date with an attractive male (priming mating motives, *N* = 47); competing with a colleague for promotion (priming competitive motives, *N* = 44); or a short story about losing their wallet and not being able to leave the house without it (control condition designed to elicit arousal but not competitive motives, *N* = 49). The short stories (between 724 and 823 words) were identical to Griskevicius et al., who rated the stories for various qualities and confirmed that they prime the intended motives. After reading the story, participants indicated how vividly they imagined the scenario on a 1 (not at all vivid) to 7 (very vivid) scale (sensu Watkins, DeBruine, Little, & Jones, 2012). People can accurately rate the vividness of their mental imagery (Pearson, Rademaker, & Tong, 2011).

Immediately following the priming phase of the study, participants took part in a shoe-shopping task. Women were asked to imagine that they had been given a sum of money for their birthday in the form of a voucher at a designer shoe store. They were asked to imagine that they go to the shop in the knowledge that they can afford to buy anything they like. Participants were asked to think about how they would feel as they walked into the shop and placed a point on a visual analogue scale that best fit the type of shoe they would be looking for. In order to match the heel sizes used in the full range of images that were pilot-tested, the scale used the left anchor point “flat shoe (no heel)” and right anchor point “very high-heeled shoe (160 mm or over 6 inches).”

#### Data Analysis

As the visual analogue scale was set up in increments from 0 to 160 (numerical scale not visible to participant), we used these data to calculate each woman’s preference for heel height in millimeters. Scores could thus range from 0 to 160. Six women did not complete this phase of the task (*N* = 136 heterosexual women reported in analyses).

### Results

A between-subjects ANOVA on the dependent variable preference for heel height, with the factor experimental priming condition (mating motive, competitive motive, control condition) revealed no effect of experimental priming condition (*F*[2, 133] = .55; *p *= .58). Including vividness of mental imagery or participant age as covariates in the model did not alter this null finding (*F*[2, 130] = .51; *p *= .60). There was no effect of vividness of mental imagery (*F*[1, 130] = 2.05; *p *= .16) or participant age (*F*[1, 130] = 2.29; *p *= .13).

On average, across experimental conditions, women selected a shoe of 6.02 centimeters in heel height (SD= 4.23 cm, range = 0–15.9 cm), which differed significantly from zero (*t*[135] = 16.59; *p *< .001, *d *= 1.42, 95% CI [53.02, 67.37]). Given that variability in heel preferences within the sample may explain a null effect of experimental priming condition in a between-subjects design, a final study tested for potential contextual changes in an individual woman’s preference for heel height by replicating the current study with a baseline and post-priming measure of preferred heel height.

## Study 4

### Method

#### Participants

A total of 141 heterosexual women took part in both phases (pre- and post-priming) of the online study (*M*_age_ = 60.14 years, SD= 11.97 years), thus providing complete data for analysis. Given the unexpected skew in age, and before analyzing data from this sample, a separate sample of 106 heterosexual women were recruited and took part in both phases of this study. For this sample, inclusion criteria of 18–30 years of age were set via the platform’s screening tool (*M*_age_ = 25.07 years, SD= 3.55 years), in order that the sample (younger sample, older sample) could be included as a factor in analyses. For both samples, American women were recruited via the buy responses function on surveymonkey.com, where participants can take part in research in exchange for the platform donating to charity.

#### Procedure

The study was identical to Study 3 except that we collected both a pre-priming measure of preference for heel height and a post-priming measure of preference for heel height, with identical instructions used on both occasions. In the priming phase of the study, 98 women were randomly allocated to the mating motives condition, 82 women were randomly allocated to the competitive motives condition, and 67 women were randomly allocated to our control condition.

### Results

A mixed-design ANOVA on the dependent variable preference for heel height, with the within-subjects factor experimental phase (pre-priming phase, post-priming phase) and the between-subjects factors experimental priming condition (mating motive, competitive motive, control condition) and sample (older sample, younger sample), revealed no significant effects or interactions (all *F *< 1.39, all *p *> .25), except for a main effect of sample (*F*[1, 241] = 24.25; *p *< .001, np^2^ = .10). The same pattern of results was found when vividness of mental imagery was included as a covariate in the model.

Independent-samples *t* tests to interpret this main effect revealed that the younger sample preferred higher heels (*M *= 56.15 mm, SD= 35.28 mm) than the older sample (*M *= 34.09 mm, SD= 33.01 mm; *t*[245] = 5.05; *p *< .001, *d *= 0.65). Of note, the same pattern of results was found regardless of whether the sample was included as a between-subjects factor in the model or whether it was replaced with participant age as a covariate in an analysis of pooled data (i.e., a main effect of age was still observed).

### Discussion

Our studies suggest that individual differences in women’s responses to heeled shoes can be examined using sexual selection theories. Consistent with our prediction that a high heel is a costly signal (e.g., Saad, 2013) used to *augment* female attractiveness (Guéguen, 2015; Morris et al., 2013; see also Lewis et al., 2017 for recent evidence) among effective competitors for mates (Vaillancourt, 2013), attractive women were more likely to choose higher-heeled shoes under time limit than their less attractive peers were. By contrast, women’s own attractiveness did not predict the proportion of lower-heeled shoes they chose under time limit, even though the two image sets were equivalent in attractiveness. Our first study suggests that when making quick choices about shoes, attractive women prefer a higher-heeled shoe. When our task was self-paced (Study 2a), attractive women had a stronger inclination to buy heeled shoes (both higher- and lower-heeled) than their less attractive peers did. This relationship remained when pooling data across two studies from separate online samples (Studies 2a and 2b). Moreover, in one study (Study 2b), relatively attractive women were more inclined to buy the attractive lower-heeled (85 mm) shoe when analyzing women’s responses to two very attractive shoes. Collectively, evidence (across both laboratory and online studies) that own attractiveness moderated women’s responses toward heeled shoes is consistent with our proposal that women augment, rather than compensate for, their physical attractiveness via heeled shoes.

We also provide the first empirical evidence, to our knowledge, that women’s responses to high-heeled shoes may be an accurate, albeit subtle, indicator of their sexual motivation. Consistent with predictions, when examining women’s responses to two very attractive heeled shoes, dyadic sexual desire (but not solitary sexual desire) predicted their inclination to buy an attractive higher-heeled shoe but did not predict their inclination to buy an attractive lower-heeled shoe. Indeed, the slopes of these two correlations differed significantly from one another. As women, in general, were equally inclined to buy these two items (a null effect of shoe in the ANOVA), the specific nature of this finding is noteworthy as it suggests that women with a stronger desire for sexual activity with a partner potentially trade off a lower heel for a higher heel when indicating their feelings toward two attractive heeled shoes. As we found no evidence, across two studies, that competitive attitudes toward other women predicted women’s responses to heeled shoes, our research suggests that wearing heels may function, in part, to aid female mate choice rather than intrasexual competition per se.

Finally, when directly examining women’s preference for heel height via a visual analogue scale (Studies 3 and 4), young women, when primed with the context that they had free choice to buy any shoe, preferred a heel in the range of 56–60 mm (Study 3: 95% CI [53.02, 67.37]). Contrary to our predictions, however, neither mating nor competitive motivations altered women’s preference toward a higher-heeled shoe when these motives were primed experimentally. Instead, women’s age had a noticeable effect on their preference for heel height, where our younger sample preferred shoes with a taller heel than our older sample did. These findings are still consistent with our general proposal that contexts where mating competition is more intense (Vaillancourt, 2013) predict preferences for a higher-heeled shoe. However, our findings suggest that between-women variation, rather than within-women variation, may be a better focus for research on differences in women’s responses to shoes, at least when we consider the contexts primed within the current studies.

Of note, although the women in our older sample preferred a lower heel, the average preference in this group still exceeded levels that can contribute to foot problems (> 25 mm, Menz & Morris, 2005). Thus, although we observed variation in heel preference according to age as predicted, women may still engage in “costly signaling” via heels at older ages, potentially for other functions. Although findings from one of our studies (Study 2b) suggest that older women were more inclined to buy heeled shoes than younger women when responding to our image set, this sample was limited to women of 18–30 years of age. As such, mating competition may be more intense within the older women in this age range, for example, in light of historical trends toward later average age at marriage (see Rotz, 2016 for American data). While this may suggest, tentatively, that the relationship between age and inclination to buy heels is curvilinear when measured across the lifespan, further work is required to examine these issues. We do note, however, that our findings for age differences in preference for heel height complement research on women’s responses to other apparel, where preferences for attractive (red) coloration in clothing are observed in young but not older women, when their hormone levels are associated with greater risk of pregnancy (Blake et al., 2017).

Future research may address potential limitations of the current set of studies. For example, it is unclear whether our findings generalize to other styles of footwear, and whether our current set of studies simply reflect women’s responses to designer footwear. Here, we were able to take advantage of access to a designer shoe label with good quality images and publicly available data on each item (including heel height). As our pilot data demonstrated strong correlations between attractiveness, sexiness, and heel height and strong (negative) correlations between heel height and traits such as “practicality” and “comfortable,” we were limited in the extent to which we could compare responses to higher heels with those of very low heels or flat shoes. However, this limitation was offset by our design where we controlled for attractiveness differences between the two image sets. This enabled us to make stronger claims about whether women “trade off” a higher versus lower heel in light of their own attractiveness, while controlling for the general appeal of the shoe (i.e., so that the motive to purchase should be equivalent across image sets, despite potential aesthetic differences unrelated to heel height).

As our sample was selective when indicating their preference toward a set of different shoes, researchers may also develop our work by examining responses to different styles of shoe at the extremes of attractiveness. For example, further work may compare women’s responses to two equally popular/attractive items within a larger image set. It may be possible to observe priming effects with the techniques used here when comparing responses to equally attractive shoes that differ in style or function, or by using other priming techniques designed to elicit sexual desire such as erotic images or audio. Diary-based studies of women’s actual choice or use of shoes (e.g., recorded via smartphone) may also provide a useful supplement to the current research. Indeed, further work such as this would be important to examine the extent to which different styles of shoe are worn for different functions, such as comfort versus attractiveness, at different times of the day.

As there is a distinction in fashion research between purchasing and consuming said items (see Morgan & Birtwistle, 2009 and O’Cass, 2000 for discussion), this may represent a limitation to our work. However, research on evolutionary perspectives to consumer behavior has examined, and provided converging evidence, for attractiveness enhancement via fashion and apparel in studies where women are asked to indicate their desire to purchase (e.g., Durante, Griskevicius, Hill, Perilloux, & Li, 2008; Hill et al., 2012) and desire to wear given items of fashion (Durante, Li, & Haselton, 2008). Indeed, evidence that women incur financial costs for a particular item is still of theoretical interest for understanding how people desire to present themselves to others on given occasions, even if the occasions in which such items are displayed are rare. Secondly, incurring costs in this way is still of practical interest to marketers as inclinations to buy items still have an influence on markets, by definition, compared to if that same preference were absent (e.g., analogous to the distinction between mate preference and mate choice). Given our pilot data revealed a strong negative correlation between a shoe’s attractiveness and how comfortable/practical it looks, our data also reveal that, on some level, judges negatively associate a shoe’s attractiveness with its practicality, at least when viewing items from a high-end retail chain.

The research reported here complements recent work, which demonstrates augmentation of physical appearance via male beards (Dixson et al., 2017, 2018) and female breasts (Dixson et al., 2015) where, for example, the attractiveness of these features is contingent on other aspects of morphology (see Dixson et al., 2015 for discussion). In arguing that heels are costly signals that augment female attractiveness, an unaddressed question from our work is the extent to which heels augment female attractiveness independent of their morphology or if the positive effects of heels on attractiveness are qualified by aspects of their facial or bodily morphology. Examining the contributions of facial and bodily morphology, motion, and expression to perceptions of women in heels versus flat shoes will likely shed light on these issues. Indeed, an alternate proposal where heels provide an indicator of confidence, as the wearer has to maintain upright gait and confident striding locomotion, is worthy of further study, although these traits may, in part, reflect perceptions of one’s own mate value. Of note, while prior work suggests that other items of cultural apparel such as makeup enhance attractiveness to a greater extent for (naturally) less attractive women than they do for naturally attractive women (Jones & Kramer, 2016), revealing a role for cultural apparel in compensating appearance, our findings suggest that use of heels may function for augmentation. Extending our paradigm and those used by others to investigate social judgments (e.g., Jones & Kramer, 2016) to examine responses to various forms of clothing and apparel that enhance status and/or attractiveness would likely prove fruitful if tested in both men and women. Examining the relative contributions of different fashion items and/or cosmetics to trait judgments, the importance of augmentation and appearance enhancement, and individual differences in orientation to different items can shed light on the use of cultural apparel in shaping social interactions, which may be of interest to both academics and marketers.

It is also worth noting that our research examined ultimate-level explanations (Scott-Phillips, Dickins, & West, 2011) for women’s choice of footwear (to improve attractiveness) and as such is not arguing that motives for the purchasing decisions observed in our studies necessarily reflect a conscious strategy among women. Nonetheless, our data suggest that sexual selection theories have utility for understanding shoe preference if the behavior (i.e., purchasing and displaying shoes) has a positive effect on reproductive fitness by enhancing one’s attractiveness relative to others (see also Vaillancourt, 2013 for discussion). It would, however, also be of great value to study women’s responses to shoes at the proximate level, for example, by attempting to reduce women’s cognitive and affective responses to various items of footwear to their primary dimensions via a data-driven approach (e.g., to test for divergent and/or converging evidence, see Munafo & Smith, 2018). Examining preferences for heeled shoes across generations would also likely prove fruitful, if historical data exist on this such as data from sales or observations from work of art. Of note, although data from our first study revealed no effect of self-reported height on women’s shoe choices, further work, which takes a data-driven and/or theory-driven approach, could examine the role of height and/or body size more generally in women’s shoe choices, particularly as size alters the costs versus benefits of wearing heeled shoes (e.g., Titchenal et al., 2015). Such research could consider women’s height/size in absolute terms and/or when compared to the height of other women within their immediate environment.

To conclude, our findings extend research on sartorial appearance by demonstrating that age, self-rated attractiveness, and dyadic sexual desire moderate women’s responses to heeled shoes. Purchasing and displaying heeled shoes may function for females to augment, rather than compensate for their attractiveness, and women may orient themselves differently toward footwear depending on their sexual motives and at times in the lifespan where mating competition is relatively intense.

## Electronic Supplementary Material

Below is the link to the electronic supplementary material.

Study 1: Examining Differences in Women’s Choices of Higher-Heeled and Lower-Heeled Shoes (CSV 4 kb)

Study 2a: Examining Differences in Women’s Inclination to Buy Higher-Heeled and Lower-Heeled Shoes (Focus on Mate Choice) (CSV 4 kb)

Supplementary material 3 (CSV 5 kb)

Study 2b. Examining Differences in Women’s Inclination to Buy Higher-Heeled and Lower-Heeled Shoes (Focus on Same-Sex Competition) (CSV 6 kb)

Study 3: Testing for Effects of Primed Mating Motives on Preference for Heel Height in Shoes (CSV 4 kb)

Study 4: Testing for Effects of Primed Mating Motives on Women’s Change in Preference for Heel Height in Shoes (CSV 8 kb)
